# On the momentum toward vaccine self-sufficiency in the BRICS: an integrative review of the role of pharmaceutical entrepreneurship and innovation

**DOI:** 10.3389/fpubh.2023.1116092

**Published:** 2023-10-09

**Authors:** Yongqiang Zhang, Hongbo Li, Xinglong Xu, Henry Asante Antwi

**Affiliations:** ^1^School of Management, Jiangsu University, Zhenjiang, China; ^2^Medical Insurance and Public Policy Research Center, Jiangsu University, Zhenjiang, China

**Keywords:** vaccine self-sufficiency, BRICS, integrative review, pharmaceutical entrepreneurship and innovation, momentum

## Abstract

**Background:**

With the perspicuous effect of COVID-19 on vaccine demand, academic and business interest in vaccine production in the BRICS nations (Brazil, the Russian Federation, India, China, and South Africa) has reached a crescendo. Aware of a “dark” past when the BRICS depended heavily on vaccines and pharmaceuticals from other parts of the world, academic curiosity on how the BRICS countries have leveraged vaccine self-sufficiency and become the hub of global vaccine production and supply is justifiable, especially in times of ineffable pandemics.

**Methods:**

The articles were searched from November 2020 to December 2022. Within this period, an electronic search of 13 reputable healthcare and public databases was conducted. The initial searches from the designated databases yielded a total of 3,928 articles. Then, duplicated studies were removed through a two-step process, articles without titles and abstracts were excluded, and the remaining 898 articles that met the qualification assessment criteria were evaluated for article quality.

**Results:**

The main entrepreneurial innovations that have quickened the pace of vaccine self-sufficiency in the BRICS include investment in artificial intelligence (AI), Big Data Analytics, and Blockchain technologies. These help to speed up the drug delivery process by enhancing patient identification or optimizing potential drug candidates for clinical trials and production.

**Conclusion:**

Over the past 20 years, the BRICS nations have achieved major strides in vaccine development, regulation, and production. The creation of the BRICS Vaccine Research and Development (R&D) Center will have a significant impact on vaccine cost and accessibility given the anticipated development of stronger research capability, production, and distribution technology, as well as stronger standardization to improve vaccine production quality in the near future. It is anticipated that the BRICS’ contributions to vaccine development will alter the global vaccination market and hasten the availability of vaccinations in developing nations. The challenge is turning these hopes into concrete plans of action and outcomes.

## 1. Introduction

Self-sufficiency is the ability of an individual, group of people, country, or group of countries to provide for or fulfill their need for a certain commodity or service without the need for external aid or support (1). Because of the importance of self-sufficiency, several countries have initiated a number of programs and policies to achieve self-sufficiency in one area or another. For example, in 2018, China’s President Xi Jinping urged the country’s agriculture experts to “vigorously develop” its research and technology with greater innovation in order to expedite efforts to achieve agricultural self-sufficiency (2). The African Union expressed worry in 2018 that food is becoming a political weapon and that self-sufficiency in food is Africa’s main line of defense. The Union claimed that Africa will remain vulnerable to manipulation by the wealthier nations until it acquires a meaningful level of liberation from food dependency (3). In terms of self-sufficiency in healthcare, the Economic Commission for Latin America and the Caribbean (ECLAC) published its Plan for self-sufficiency in health matters in Latin America and the Caribbean in 2021. This document details the lines of action, strategies, and proposals to strengthen Latin American countries’ capacities to produce and distribute vaccines and medicines in the region.

On the eve of the 2012 BRICS Summit in Fortaleza (Brazil), the BRICS resolved to champion greater South–South collaboration and technical support to increase capacity and self-sufficiency in the health sector (4). In the midst of the calamitous consequences of the COVID-19 pandemic, the BRICS initiated policies toward vaccine self-sufficiency with the establishment of the BRICS Vaccine Research and Development (R&D) Center to engage in vaccine joint research, plant co-construction, authorized local production, and mutual recognition of standards (5). The BRICS countries jointly proposed an initiative to strengthen vaccine cooperation to ensure the accessibility and affordability of vaccines in their respective countries through their equitable distribution to achieve sufficiency in producing quality vaccines (6).

Besides the impact of COVID-19, another reason why the BRICS countries have been particular about vaccine self-sufficiency is that the region is most affected by infectious diseases. Approximately 30% of children at risk of soil-borne worms globally are in BRICS countries, while 50% of children at risk of lymphatic filariasis live in India (7). The existence of these infectious diseases poses a serious threat to the survival and development of BRICS countries.

In 1993, vaccines were being produced in all five countries, but the processes were archaic and unreliable, there was little relevant research, and the products received little recognition abroad. Over the past 34 years, BRICS countries have grown from 0% of global vaccine production to 37%, according to statistics (8). The current situation shows that BRICS countries are gradually maturing in vaccine production for infectious diseases despite challenges in terms of quality.

The outbreak of COVID-19 has significantly increased the global demand for infectious disease vaccines and the BRICS countries have seized the opportunity to take a leading role in the discussion, research, development, production, and distribution of vaccines and other medical products (9, 10).

Vaccine research and development is costly and risky, with an average time from research and development to commercialization of 10 years. Within this period, market conditions, technology, and capital markets can change drastically, leading to a futile venture (11). Because of these difficult-to-control external pressures, average-sized biomedical companies are afraid of vaccine research and development. This notwithstanding, all five BRICS countries have developed strong initiatives for the development of vaccine technology and greatly improved their national regulatory capacity to improve vaccine production. As of August 2019, BRICS countries accounted for 25% of global biotechnology patent registrations, 35.7% of pharmaceutical patent registrations, and 18.3% of medical technology registrations (12–18). This information also confirms that healthcare innovation is increasing in BRICS countries.

According to Singh and Chattu (13), the revolution in vaccine and pharmaceutical research and production is not taking place in a vacuum but through complex and tortuous channels. The road to vaccine self-sufficiency has often been one of vitriolic criticism from skeptics, critics, and competitors, yet there is a determination to turn the situation around (14–17). Therefore, facing the rise of the vaccine production industry in BRICS countries, it is necessary to conduct exploratory analysis and research on the development of the vaccine industry in BRICS countries.

Although the current situation of the enhancement of infectious disease vaccine production capacity in BRICS countries is obvious, few scholars have reviewed and analyzed the process of the improvement of infectious disease vaccine production capacity. Therefore, we need to systematically review the dynamics of the BRICS vaccine industry to achieve these developments and achieve self-sufficiency. This paper will explore and synthesize knowledge on the contribution of entrepreneurship and innovation to vaccine self-sufficiency for infectious diseases in BRICS countries, based on a theoretical perspective on entrepreneurship. It also relies on Lumpkin and Dess (15) to conceptualize entrepreneurial orientation (innovation, risk-taking, competitiveness, and autonomy), extends Lumpkin and Dess’s (15) individual-level entrepreneurial orientation dimension to understanding national entrepreneurial orientation and behavior, and analyzes the process of vaccine self-sufficiency in BRICS countries.

Based on this, the main purpose of this study is to explore how the BRICS countries can promote vaccine self-sufficiency in BRICS countries. By exploring the answer to this question, we can provide some experience and lessons for the vaccine production path of other countries.

## 2. Methods

This study uses a comprehensive research approach relative to other heavily scrutinized methods, as the inclusion of studies with different approaches and larger contexts is essential if one is to better understand the vaccine industry where politics and healthcare converge (19). Although the global health industry as a whole is complex, the vaccine component of the industry is highly manipulated and shaped by political polarization and economic, sociocultural, ecological, and technological factors. Moreover, the industry is seamlessly integrated with regulatory practices that cross borders because of its sensitivity.

**Figure 1:**
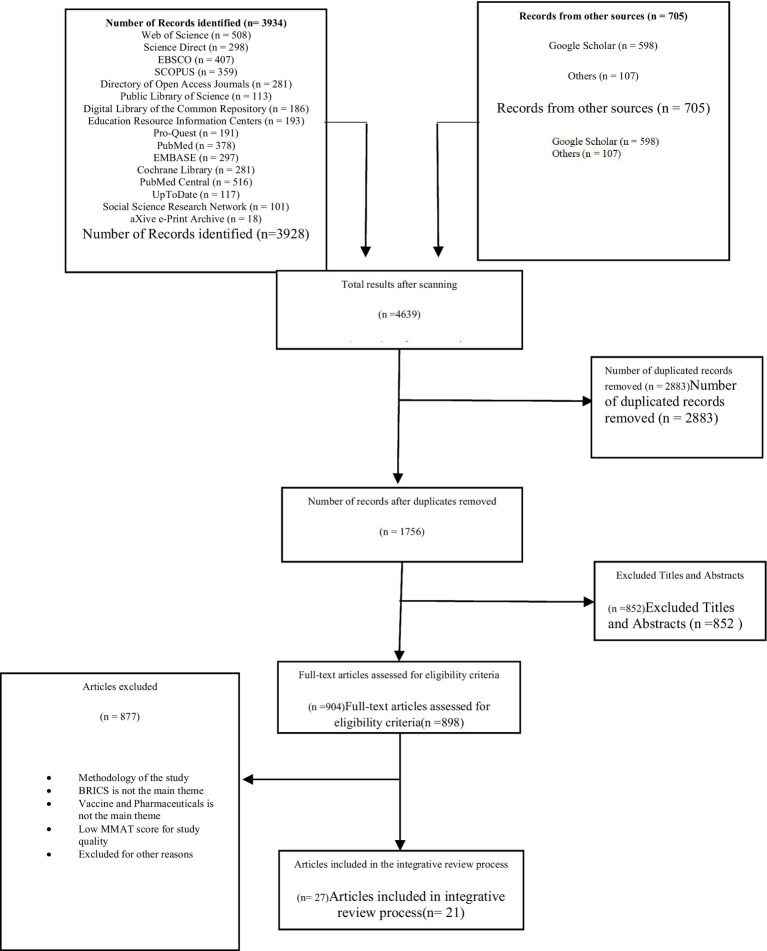
Flow of article selection process.

Thus, an integrated approach allows researchers to better distinguish fact from fiction. In addition, the comprehensive retrospective approach is used in this study because it has better potential to simply reveal facts without compromising informed research, systems analysis, theoretical applications, and the direct applicability of the results to practice and policy development (20). The systematic literature review method is an analysis method based on the collection and collation of existing literature, which researches and excavates corresponding fields in accordance with systematic steps to find the existing research focus, direction, and problems in this field. This method embodies the characteristics of objectivity, clarity, rigor, and openness of scientific research. It is generally believed that Tranfield et al. (9) first proposed the systematic literature review method. Figure 1 shows the sequence of activities proposed by Souza et al. that must be performed in a good comprehensive review (21). In addition, guiding questions are first defined and literature retrieval and sampling, data collection for included literature, critical analysis, discussion of results, and presentation of a comprehensive review are conducted. In addition, we used the Preferred Reporting Project (PRISMA) checklist for systematic review and meta-analysis to ensure that the scientific literature being reviewed was not arbitrarily selected. These steps are examined in turn below.

In addition, in order to verify the quality of the selected article and ensure its scientific and applicable nature of the research. The quality of the selected articles was evaluated using the time-tested Mixed Methods Appraisal Tool (MMAT), as presented in Table 1. Pluye and Hong argue that the MMAT quality appraisal indices accommodate studies undertaken with different methodologies and strategies. This is particularly important in this integrative research composed of a maze of experimental, non-experimental, qualitative, quantitative, mixed, primary, and secondary research methods.

**Table 1:** Mixed Methods Appraisal Tool (MMAT) study quality evaluation schedule.

Study	Clarity of research questions	Relevance of data sources	Relevance of data analysis process	Relevance of findings to context?	Clarity of sampling process	Relevance of sampling technique	Representativeness of sample	Risk of researcher bias	Reasons for study target	Adequate interpretation of outcome	Risk of influence of funder	Total
Bond and Garcia	Y	Y	Y	Y	Y	Y	Y	L	Y	Y	L	100%
Chattu et al.	Y	Y	Y	Y	Y	Y	Y	L	Y	Y	L	100%
Duijzer et al.	Y	Y	Y	Y	Y	Y	Y	L	Y	Y	L	100%
Ezziane	Y	Y	Y	Y	Y	Y	Y	L	Y	Y	L	100%
Fonseca et al.	Y	Y	Y	Y	Y	Y	Y	L	Y	Y	L	90%
Guimarães	Y	Y	Y	Y	Y	Y	Y	L	Y	Y	L	100%
Hayman et al.	Y	Y	Y	Y	Y	Y	Y	L	Y	Y	L	100%
Chirmule	Y	Y	Y	Y	Y	Y	Y	L	Y	Y	L	100%
Kaddar et al.	Y	Y	Y	Y	Y	Y	Y	L	Y	Y	L	100%
Lee et al.	Y	Y	Y	Y	Y	Y	Y	L	Y	Y	L	100%
Lin et al.	Y	Y	Y	Y	Y	Y	Y	M	Y	Y	M	80%
Nhamo	Y	Y	Y	Y	Y	Y	Y	L	Y	Y	L	90%
Zoshchouk	Y	Y	Y	Y	Y	Y	Y	L	Y	Y	L	90%
Possas et al.	Y	Y	Y	Y	Y	Y	Y	L	Y	Y	L	100%
Rahalkar et al.	Y	Y	Y	Y	Y	Y	Y	M	Y	Y	L	90%
Singh and Chattu	Y	Y	Y	Y	Y	Y	Y	M	Y	Y	L	90%
Podolskaya et al.	Y	Y	Y	Y	Y	Y	Y	L	Y	Y	L	100%
Su et al.	Y	Y	Y	Y	Y	Y	Y	L	Y	Y	L	100%
Glover, et al.	Y	Y	Y	Y	Y	Y	Y	L	Y	Y	L	100%
Xu et al.	Y	Y	Y	Y	Y	Y	Y	M	Y	Y	M	80%
Yueqin	Y	Y	Y	Y	Y	Y	Y	M	Y	Y	M	80%
Sekhejane	Y	Y	Y	Y	Y	Y	Y	L	Y	Y	L	100%
Moore	Y	Y	Y	Y	Y	Y	Y	L	Y	Y	L	100%
de Paula Bueno	Y	Y	Y	Y	Y	Y	Y	L	Y	Y	L	100%
Zondi	Y	Y	Y	Y	Y	Y	Y	L	Y	Y	L	100%
Xu	Y	Y	Y	Y	Y	Y	Y	L	Y	Y	L	100%
Arup	Y	Y	Y	Y	Y	Y	Y	M	Y	Y	M	80%

Y, Yes (10 Points); L, Low (10 points); M, Medium 5 points.

### 2.1. Search strategy

The study period for this research was divided into two. The first set of articles was searched between November 2020 and November 2021. An additional set of new articles was searched between December 2021 and December 2022 to obtain more recent information. Within this period, an electronic search of 13 reputable healthcare and public databases was conducted. These were Web of Science, Science Direct, EBSCO, SCOPUS, Directory of Open Access Journals, Public Library of Science, Digital Library of the Commons Repository, Education Resources Information Center, Pro-Quest, PubMed, EMBASE (Excerpta Medica Database), Cochrane Library, PubMed Central (PMC), and UpToDate.

The study also searched for articles from major healthcare hubs, such as the databases of the Johns Hopkins University, World Health Organization, John Snow Labs, Dateva – Health Data to Health Insights, Apixio, Decision Resources Group, Optum, The Economist Intelligence Unit, Refinaria de Dados, Virtusa vLife, Medisafe, and Advera Health Analytics. The authors defined distinct and hierarchical search cluster terms to query the databases, i.e., main topic, sub-topic, and specific theme. Each article was selected through a narrative search. The key search terms were “vaccine production, pharmaceutical production, essential drug production, active pharmaceutical ingredients, global vaccine production, BRICS vaccine production, pharmaceutical patent, pharmaceutical/vaccine export, and import.” The various search terms were combined with Boolean operators AND (to narrow the search results to include only key-termed results that contain the required keywords), OR (to expand the search results to contain at least one or more of the defined key terms and phrases), NOT (to limit the search results), Quotation Marks “”, (to limit the query the system to return in results in the exact order) and Parentheses (to give priority to results containing specific keywords over other elements around it). The search terms were entered individually in English.

We further employed truncations and wildcard characters to broaden the word search and improve the sensitivity and precision of searches. The authors did not discriminate between articles based on research design (primary/secondary research, qualitative/quantitative research, essay, dissertation, or experimental/non-experimental paper). The authors limited articles of interest to those published between January 2001 and December 2022. This was to make sure that more recent information about vaccine development in the BRICS was selected and analyzed to obtain robust conclusions. The initial searches from the designated databases yielded a total of 3,928 articles. Cross-checks supplemented these from their respective reference lists and additional searches on Google Scholar and other databases to widen the article range. Through this process, 705 additional articles were retrieved and added to the selection process.

Finally, the quality of the selected articles was evaluated using the time-tested Mixed Methods Appraisal Tool (MMAT), as presented in Table 1. Pluye and Hong (10) argue that the MMAT quality appraisal indices accommodate studies undertaken with different methodologies and strategies. This is particularly important in this integrative research composed of a mix of experimental, non-experimental, qualitative, quantitative, mixed, primary, and secondary research methods. When evaluating the quality of the selected articles using the MMAT, we mainly selected the following evaluation elements to test the quality of the articles: clarity of research questions; relevance of data sources; correlation of data analysis process; how the survey result relates to the background; clarity of sampling process; correlation of sampling techniques; representativeness of samples; risk of researcher bias; reasons for research objectives; full interpretation of the results; impact risk of the contributor. By introducing the rating of each standard in detail, we can better understand the quality of the included study (16).

### 2.2. Eligibility

We set strict criteria to include and exclude articles for final analysis. The first condition was that the article must be published in the English language. This condition is necessary because it makes it easier to cross-reference with other articles that have cited it and to verify the accuracy of the article’s citation. The second criterion was that the title and theme of the article should address the topic-specific issue of vaccine production in Brazil, Russia, India, China, and South Africa. Articles that focused on the domain area of pharmaceutical production or production of active pharmaceutical ingredients were also admitted if they focused on the BRICS or compared the global production trends where the BRICS countries were discussed. Thirdly, peer-reviewed articles were prioritized, and where the article did not meet this criterion, it should have been published by a recognizable healthcare authority such as the World Health Organization (WHO), International Health Research Institutions such as Johns Hopkins University, and Centres for Disease Control (CDC), etc. The author determined whether the articles were included for full-text analysis with the assistance of trained literature search specialists based on pre-determined eligibility criteria. Publications that were disputable were further validated through a snowballing of other relevant considerations and deliberations among the research team members until a consensus was reached to accept or reject its inclusion.

### 2.3. Data extraction and analysis

For the articles that met the requirements after screening through the above process, we further adopted the full-text analysis method for testing. We first evaluated the quantitative indicators in each article. These included the analysis of observed measurable data, representative graphs, projected graphs, production and sales volumes, production and sales values, ratios, export and import values, count of production facilities, number of vaccines administered within time frames, and other quantifiable information were extracted and compared through horizontal analysis and common size analysis. The qualitative details were removed and synthesized using a four-step content analysis procedure. The articles were initially scanned for conceptual thoughts and relevant perspectives on vaccine production and pharmaceutical self-sufficiency in the BRICS. These were extracted and coded inductively by the research team.

Overlapping concepts, thoughts, and perspectives across articles were included once. Thirdly, the qualitative data were then extracted into a matrix and rigorously checked to ensure the uniqueness of each item entered into the matrix. The matrix also helped to determine the relationship between the different thoughts and their source of origin to ensure that single-sourced information was not duplicated unnecessarily. Finally, the articles’ research design and methodological quality were assessed.

The selection flow of the reviewed articles is presented in Figure 1. In this diagram, the number of articles selected at each stage of the research process is illustrated using the integrative research steps in tandem with the PRISMA steps to ensure a robust data collection procedure.

### 2.4. Screening

The first step in the screening process was to remove duplicated studies through a two-step process. The articles were extracted to four citation managers, namely Endnotes, Mendeley, Zotero, and Sciwheel, simultaneously, to reconcile the count of articles in each citation. The authors and four well-trained senior researchers in library and archival reference management systems removed all duplicated articles. After screening the articles, the content of the four citation managers was manually inspected and compared in turn by all four teams to ensure the similarity of observed content. After this stage, 2,883 duplicated articles were removed from the dataset. The remaining 1750 articles qualified to enter the next phase of the selection criteria. After that, articles without titles and abstracts were further excluded, and the remaining 898 articles that met the qualification assessment criteria were evaluated for article quality.

The screening process was conducted using the COVIDENCE software. Two authors independently screened the abstracts, and articles with absolute consensus were passed on to the full-text screening or dropped. Where there was a conflict between the authors, a third author was assigned to review the article and to break the tie to either accept or reject the abstract. The full-text screening also involved two independent reviewers. Each read the full manuscript independently and determined whether they qualified to be included in the final articles to be extracted. Where there was no consensus, a third reviewer was assigned to review it and break the tie as to whether it should be included or excluded from the final count of articles for extraction.

### 2.5. Study quality

The MMAT score of 14 papers was above 90%, and 2 articles obtained an MMAT score of 80%. Most of the articles received a score of 100% concerning the relevance of sources, the relevance of the data analysis process, the relevance of findings to context, and the clear description of the sampling process. The weaknesses of the articles that did not obtain a full score were surrounding the potential influence of the researcher and the funding agency’s role in the study.

## 3. Results

### 3.1. Study characteristics

The characteristics of the selected articles are presented in Table 2. Approximately 11% of the articles attributed the development of the BRICS vaccine industry to the innovation policies of these countries, which are rapidly achieving global self-sufficiency in drugs and vaccines. Stretsova and others all believe that the biotechnology and related patent policies of BRICS countries are important factors in promoting the innovation and development of the vaccine industry in these regions. Chattu et al. not only recognized this point but also proposed that the outbreak of COVID-19 has also objectively promoted the innovation of the BRICS vaccine industry. Wilston et al. believe that innovation support policies from the government play an important role. The publication dates of these articles range between 2014 and 2022. The second set of articles deals with general pharmaceutical production capabilities and competencies in the BRICS. Some of the articles also compare the BRICS with the global picture.

**Table 2:** Characteristics of the selected articles.

Study	Year of publication	Thematic area	Focus of study	Study source country
Chattu et al. (22)	2021	Innovation policy	Patent and COVID-19	India
Podolskaya et al. (23)	2021	Innovation policy	Biotechnology patenting in the BRICS	Russia
Glover et al. (24)	2021	Innovation policy	Policies that encourage innovation	England
Bond and Garcia (25)	2020	Pharmaceutical production	BRICS transformative strategy	Spain
Ezziane (14)	2014	Pharmaceutical production	Essential drugs production in the BRICS	South Africa
Lee et al. (26)	2021	Pharmaceutical production	Funding pharmaceutical laboratories in the BRICS	China
Rahalkar et al. (2)	2021	Pharmaceutical production	Challenges of biopharmaceutical industry in the BRICS	India
Yueqin (27)	2020	Pharmaceutical production	Cooperation among the BRICS countries	China
Fonseca et al. (28)	2020	Vaccine production	The BRICS response to COVID-19	Portugal
Guimarães (29)	2021	Vaccine production	Vaccines business	Brazil
Hayman et al. (30)	2021	Vaccine production	Innovation for vaccine manufacturers	Brazil
Chirmule (31)	2021	Vaccine production	Technology transfer in human vaccinology	England
Kaddar et al. (10)	2014	Vaccine production	BRICS investment in vaccine development	Israel
Nhamo (18)	2021	Vaccine production	COVID-19 vaccines development discord	South Africa
Zoshchouk (32)	2021	Vaccine production	China in the global vaccine market	Australia
Possas et al. (19)	2021	Vaccine production	Vaccine innovation	South Africa
Xu et al. (33)	2014	Vaccine production	Chinese vaccine products in the global market	China
Gadelha et al. (34)	2020	Vaccine supply chain	Vaccine supply chain	Netherlands
Lin et al. (35)	2021	Vaccine supply chain	Cold chain transportation	China
Singh and Chattu (6)	2021	Vaccine supply chain	Equity’in COVID-19 vaccine distribution	India
Su et al. (7)	2021	Vaccine supply chain	COVID-19 vaccine donations	China
Sekhejane (36)	2023	BRICS cooperation	Vaccine production potential	South Africa
Moore (20)	2023	BRICS health cooperation	Vaccine diplomacy	Brazil
de Paula Bueno (37)	2023	BRICS health cooperation	Vaccine diplomacy	China
Zondi (38)	2023	BRICS health cooperation	BRICS health cooperation	Brazil
Xu (39)	2023	BRICS health cooperation	Intra-BRICS cooperation	China
Arup (40)	2023	BRICS health cooperation	Patents and other conditions of access to vaccines	India

There are also articles that focus on Pharmaceutical Production. The dates of publication of the articles range between 2014 and 2022 and constitute 19% of the reviewed articles. Bond, P. and Garcia, A believe that the national change strategies of BRICS countries are reflected in the field of drug production. According to Ezziane Z, the production level of essential medicines in BRICS countries, namely basic drug research and development and production capacity, is also an important factor in determining the development of the vaccine industry. Lee and Rahalka, among others, focus on pharmaceutical research and development, arguing that it is vital to increase the number of national laboratories and address the challenges facing the biopharmaceutical industry. Yueqin makes an interesting observation that the study sees cooperation among BRICS countries as a driving force for innovation in their vaccine industries. Articles on the vaccine supply chain accounted for 15% of the total number of articles reviewed, with one article focusing on vaccine production and supply chains in the BRICS countries and elsewhere including the largest number of publications analyzed in this study. Approximately 33% of the articles examined vaccine production in BRICS countries and how BRICS countries have become self-sufficient in vaccines over the past decade from different perspectives, including COVID-19 response, vaccine business expansion, technology transfer, investment in vaccine development, and countries’ positions in the global vaccine market. By 2023, the focus of relevant research obviously shifted to health coordination among BRICS countries, and the number of articles in this part accounts for 22%.

These articles examine global vaccine production (including in the BRICS) before and during the COVID-19 pandemic and predictions beyond COVID-19. Approximately 38% of the articles used qualitative research methods, whereas 9% employed quantitative research methods. The remaining articles were conducted with a mixed methodology. Most of the studies were conducted based on the analysis of secondary data or a review of previous studies. This is a common practice in the healthcare sector, where official sources are trusted sources for official data. Approximately 66% of the studies were focused on the BRICS, whereas 34% had a global focus. Even though the settings of the studies were largely BRICS countries, the backgrounds of the authors widely varied across all continents.

The proportion of pre-qualified vaccines produced in a particular country is a perfect measure of vaccine self-sufficiency. This pre-qualification requires the new vaccines to thoroughly evaluate relevant data testing of samples and WHO-pertinent manufacturing inspection locations. If it is satisfied with the process, it declares a vaccine to meet the responsible WHO unit’s safety, quality, and efficacy standards. It also indicates that the vaccines meet the operation specifications for the packaging and presentation of United Nations institutions that want to procure vaccines. Table 3 shows the percentage of pre-qualified vaccines produced in the BRICS by 2013. This table shows that by 2012, pre-qualified vaccine production in the BRICS had picked up. At that point, the BRICS was home to nearly 40% of the global population. In 2020, the BRICS regions produced the highest number of pre-qualified vaccines worldwide. In 2020, 46 manufacturers of pre-qualified vaccines bought the World Health Organization, of which the BRICS regions are home to 17, and they represent 37% of the total manufacturing base of the global production of pre-qualified vaccines.

**Table 3:** Global production of pre-qualified vaccines for the BRICS (1986–2021).

Year	Number of vaccines	Number of manufacturers	BRIC vaccine manufacturing		% From BRICS
			Number of BRICS manufacturers	Institutions and vaccines produced	% From BRICS
1986	6	13	0		0%
1996	13	18	1	India-Serum Institute of India (DT, DTP, M, Td, TT)	6%
2006	24	22	8	Brazil-Bio-Manguinhos (YF), India-Biological E. (TT), India-Cadila Health Care (rabies), India-Chiron Behring Vaccines (rabies), India-Haffkine Bio Pharmaceutical Corporation (OPVa), India-Panacea Biotec (OPVa), India-Serum Institute of India (BCG, DT, DTP, DTP–hep B, hep B, M, MR, MMR, rubella, Td, TT), India-Shantha Biotechnics (hep B)	36%
2012	33	27	9	Brazil-Bio-Manguinhos (BMPa, YF), India-Bharat (hep B,b OPVa,b), India-Biological E. (Pent., TT), India-Cadila Health Care (rabies), India-Chiron Behring Vaccines (rabies), India-Haffkine Bio Pharmaceutical Corporation (OPV 1–3,a OPV 1,a OPV 1 + 3a), India-Panacea (DTP–hep B,b hep B,b OPV,a,b OPV 1 + 3a,b, Pent.b), India-Shantha Biotechnics (C, hep B,b Pent.,b TT), India-Serum Institute of India (BCG, DT, DTP, DTP–hep B, hep B, Hib, M, meningococcal A conjugate, MR, MMR, pandemic influenza, Pent., rubella, Td, TT), Russia-Chumakov Institute of Poliomyelitis and Viral, Russia-Encephalitides (YF)	33%
2013	33	34	10	Brazil-Bio-Manguinhos (BMPa, YF), India-Bharat (hep B,b OPVa,b), India-Biological E. (Pent., TT), India-Cadila Health Care (rabies), India-Chiron Behring Vaccines (rabies), India-Haffkine Bio Pharmaceutical Corporation (OPV 1–3,a OPV 1,a OPV 1 + 3a), India-Panacea (DTP–hep B,b hep B,b OPV,a,b OPV 1 + 3a,b, Pent.b), India-Shantha Biotechnics (C, hep B,b Pent.,b TT), India-Serum Institute of India (BCG, DT, DTP, DTP–hep B, hep B, Hib, M, meningococcal A conjugate, MR, MMR, pandemic influenza, Pent., rubella, Td, TT), Russia-Chumakov Institute of Poliomyelitis and Viral, Russia-Encephalitides (YF)	29%
2021		46	17	China-Sinovac Biotech Company Ltd. (COVID), China-Beijing Institute of Biological Products Company, Brazil-Bio-Manguinhos (BMPa, YF), India-Bharat (hep B,b OPVa,b), India-Biological E. (Pent., TT), India-Cadila Health Care (rabies), India-Chiron Behring Vaccines (rabies), India-Haffkine Bio Pharmaceutical Corporation (OPV 1–3,a OPV 1,a OPV 1 + 3a), India-Panacea (DTP–hep B,b hep B,b OPV,a,b OPV 1 + 3a,b, Pent.b), India-Shantha Biotechnics (C, hep B,b Pent.,b TT), India-Serum Institute of India (BCG, DT, DTP, DTP–hep B, hep B, Hib, M, meningococcal A conjugate, MR, MMR, pandemic influenza, Pent., rubella, Td, TT), Russia-Chumakov Institute of Poliomyelitis and Viral, Russia-Encephalitides (YF), China-Hualan Biological Bacterin Inc. (COVID), Brazil- Institutto Butantan (COVID), China-Sanofi Health India Private Ltd. (COVID), China-Xiamen Innovax Biotech Ltd. (COVID)	37%

Source: WHO – Prequalification of Medical Products (IVDs, Medicines, Vaccines and Immunization Devices, Vector Control), 2021.

**Figure 2:**
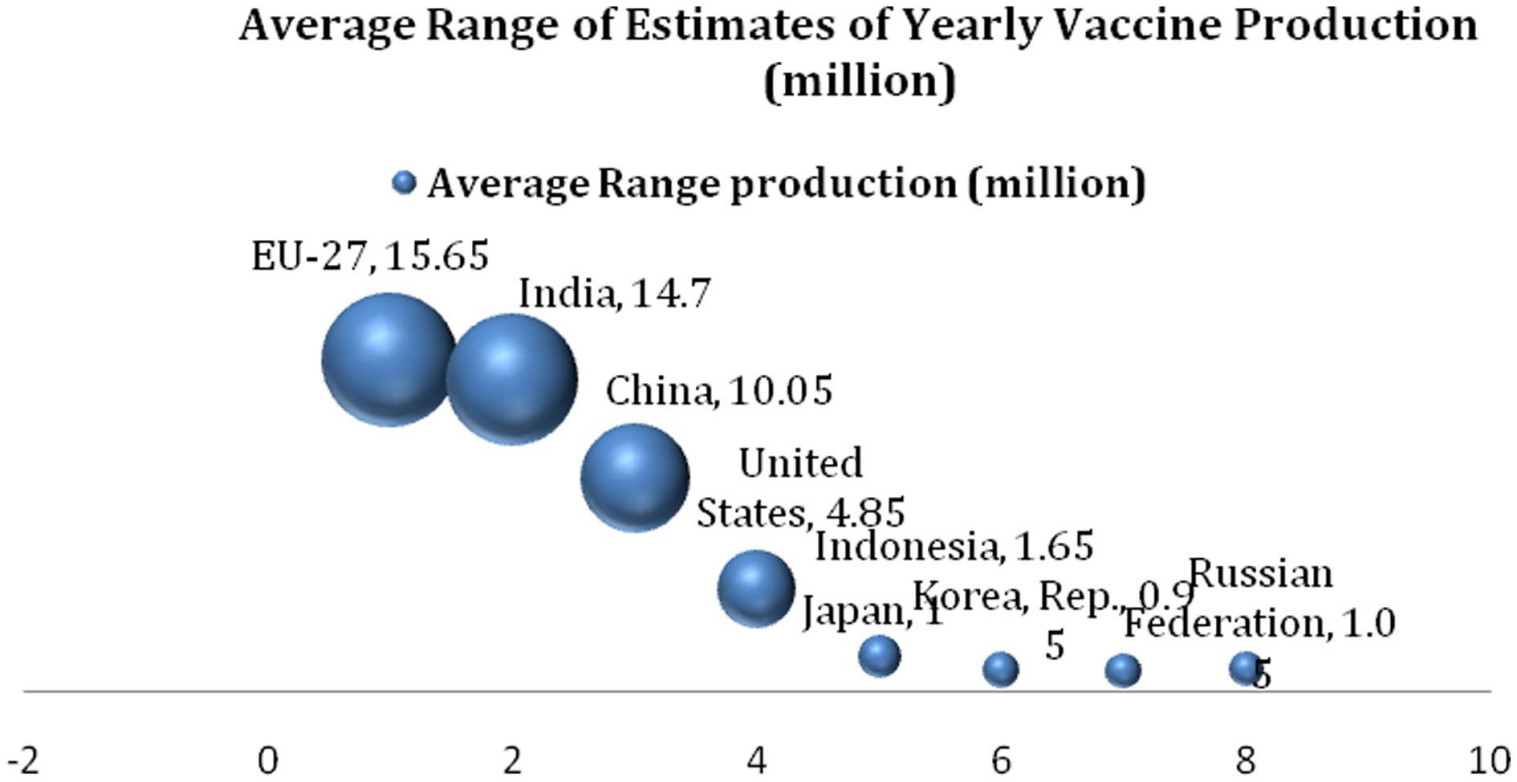
Range of estimates of yearly vaccine production (2017–2019).

Figure 2 provides details of the ranking of top countries and regions in the vaccine production industry between 2017 and 2019, just before the COVID-19 pandemic. Together, these countries (regions) produce approximately 95% of global vaccines, and the EU bloc leads with an average production capacity of 15.65 million vaccines *per annum*. India closely follows this, with a production capacity of 14.7 million vaccines, alongside China, which was shown to have produced 10.05 million vaccines on average each year from 2017 to 2019. The United States produced approximately 4.85 million vaccines within the period, while Indonesia and the Russian Federation produced 1.65 million vaccines and 1.05 million vaccines, respectively. Japan and the Republic of Korea follow, respectively, with the production of 1 million vaccines and 0.95 million vaccines. Supposing the vaccine production capacity among the BRICS members is accumulated, it is revealed that the BRICS (China, India, and Russia) produced a total of 25.8 million vaccines, which is more than half of the countries that produce 95% of global vaccines.

**Figure 3:**
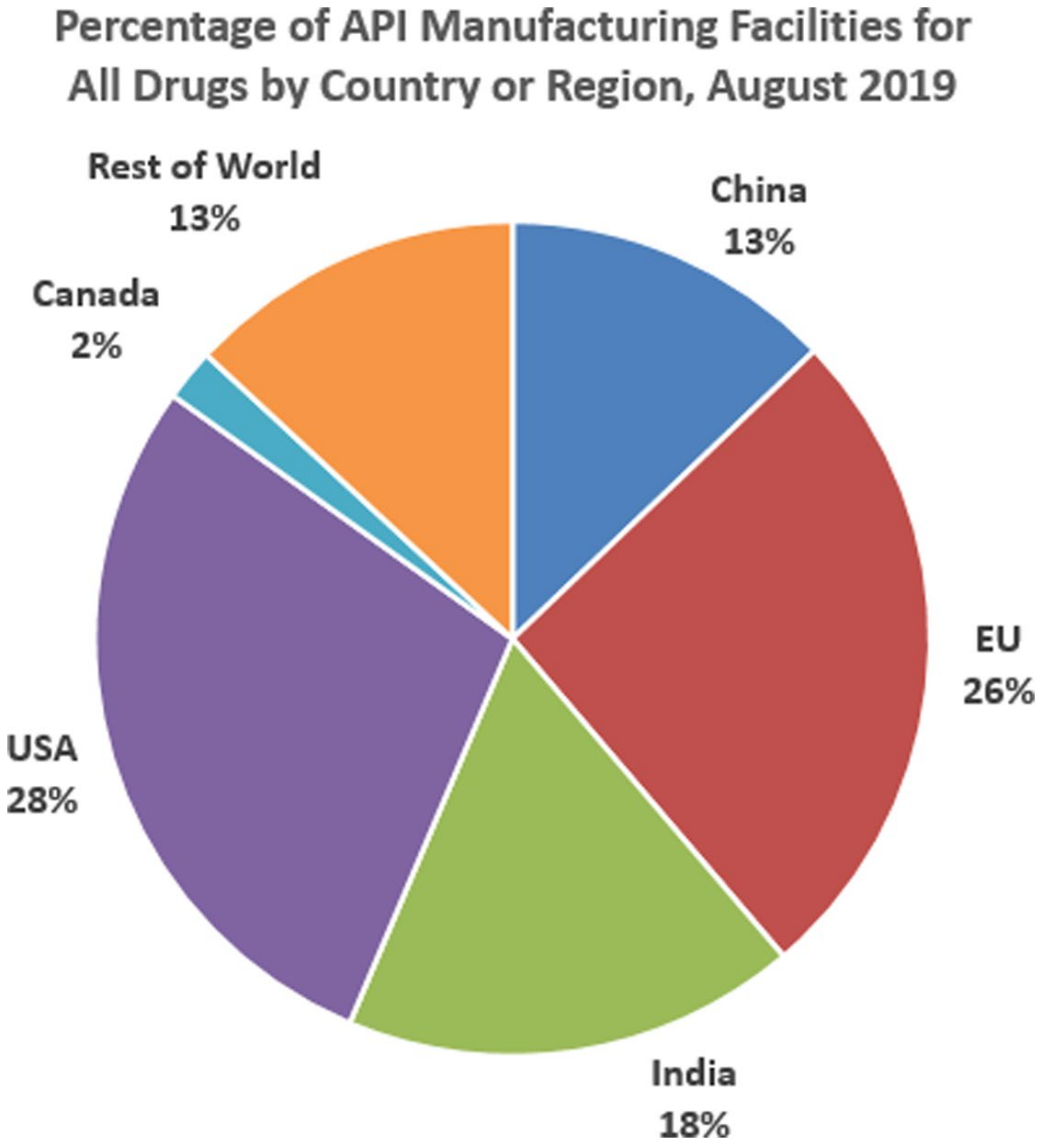
Manufacturing sites of APIs for U.S. market by country or region, August 2019.

Figure 3 presents the percentage locations of Active Pharmaceutical Ingredients Manufacturing Facilities for all drugs in the US healthcare market. Active Pharmaceutical Ingredients are important because they are the substances used in the production of finished pharmaceutical products with the intent of furnishing pharmacological activity or otherwise having a direct effect on the diagnosis, cure, mitigation, treatment, prevention, correcting, modifying, and restoring health in one form or another. Historically, the production of medicine for the US market has been done domestically, but this trend has changed, and major drug manufacturing companies have relocated their production hubs to other destinations for both business and practical reasons. This is particularly the case for active pharmaceutical ingredients. Again, the BRICS countries have been a significant contributor to the active manufacturing of pharmaceutical ingredients, as shown above. For example, while the EU manufactures 26% of active pharmaceutical ingredients consumed in the US, India ranks second with a percentage manufacturing rate of 18%. China (13%), Canada (2%), and the rest of the world (13%) follow in that order. This implies that the BRICS members represented in the chart (China and India) produce 31% of all Active Pharmaceutical Ingredients consumed in the US, which outperforms those produced in the north (America, USA, and Canada, 30%), the EU (26%), and the rest of the world (13%).

**Figure 4:**
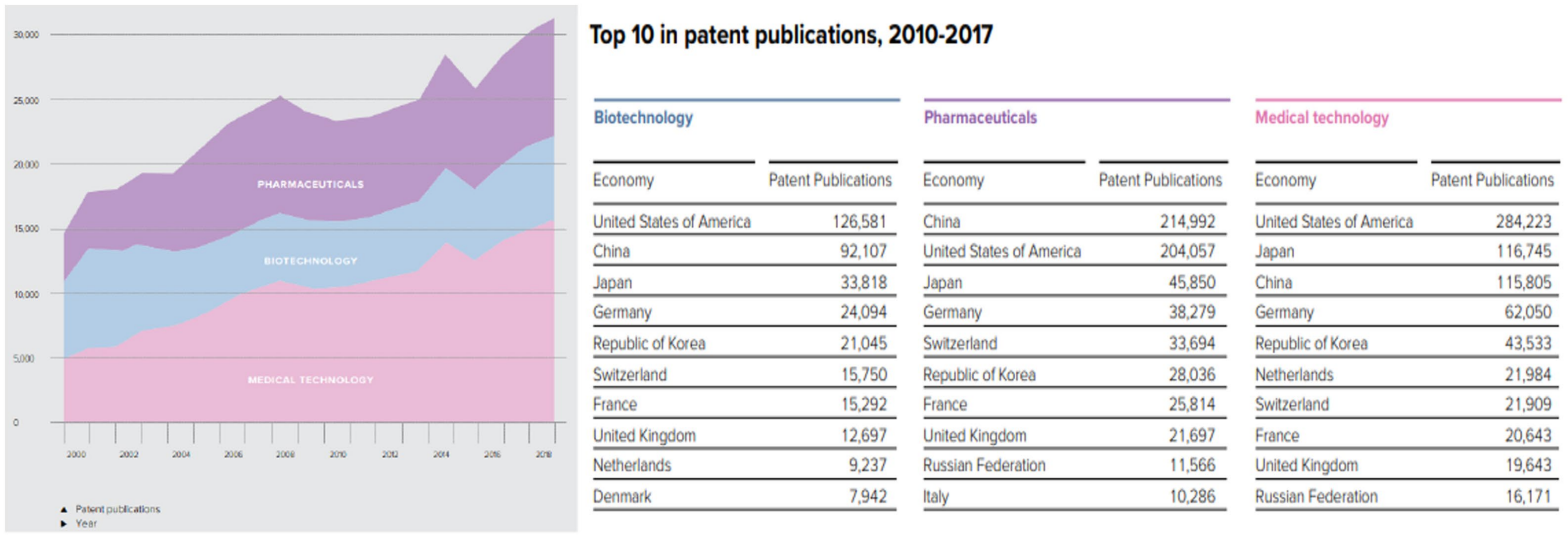
Destination for top 10 patent registration industries in the health sector.

The number of registered patents is a test of the innovation capability of a country or a firm. In Figure 4, the top 10 patent registration industries in the health sector globally are presented. Medical technology, pharmaceuticals, and biotechnology patents are the fastest registered patents within the period. The top 10 countries registering these patents are shown in Figure 4. China and Russia are the only BRIC countries represented and account for nearly 25% of biotechnology patent registration, 35.7% of pharmaceutical patent registration, and 18.3% of medical technology. This information affirms that the geography of medical innovation is shifting, though moving toward emerging economies such as China, India, and Mexico.

**Figure 5:**
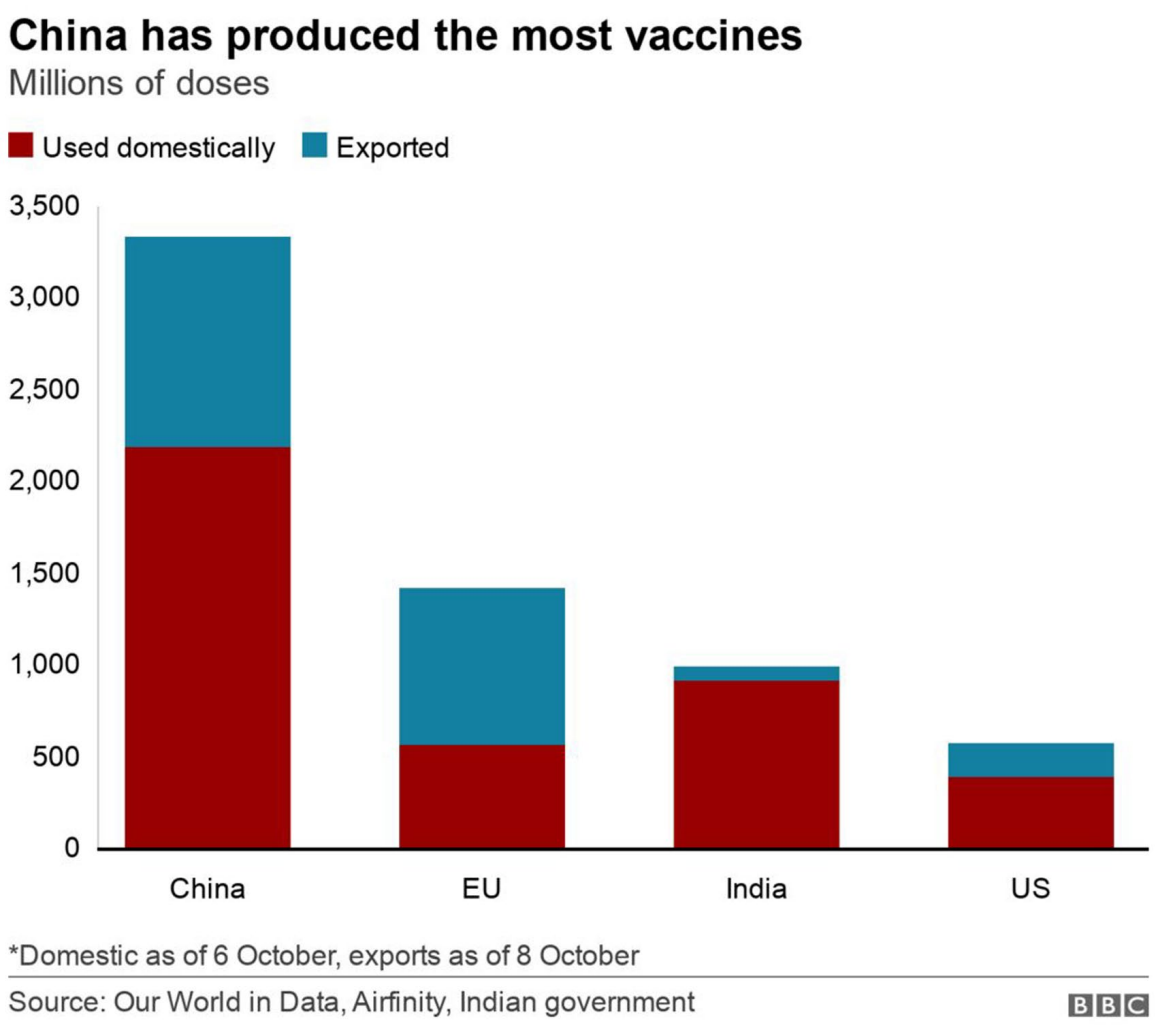
Global production of vaccines for domestic use and export.

Figure 5 shows the production and export of COVID-19 vaccines for respective countries and blocs. The analysis shows that as of October 2021, China alone has produced and exported more vaccines than the combined production of the US, the EU, and even India. If considered in a bloc, the contribution of the BRICS in this data (China and India) is far greater than the production and export of COVID-19 vaccines. This information further testifies to the vaccine production leadership of the BRICS.

**Figure 6:**
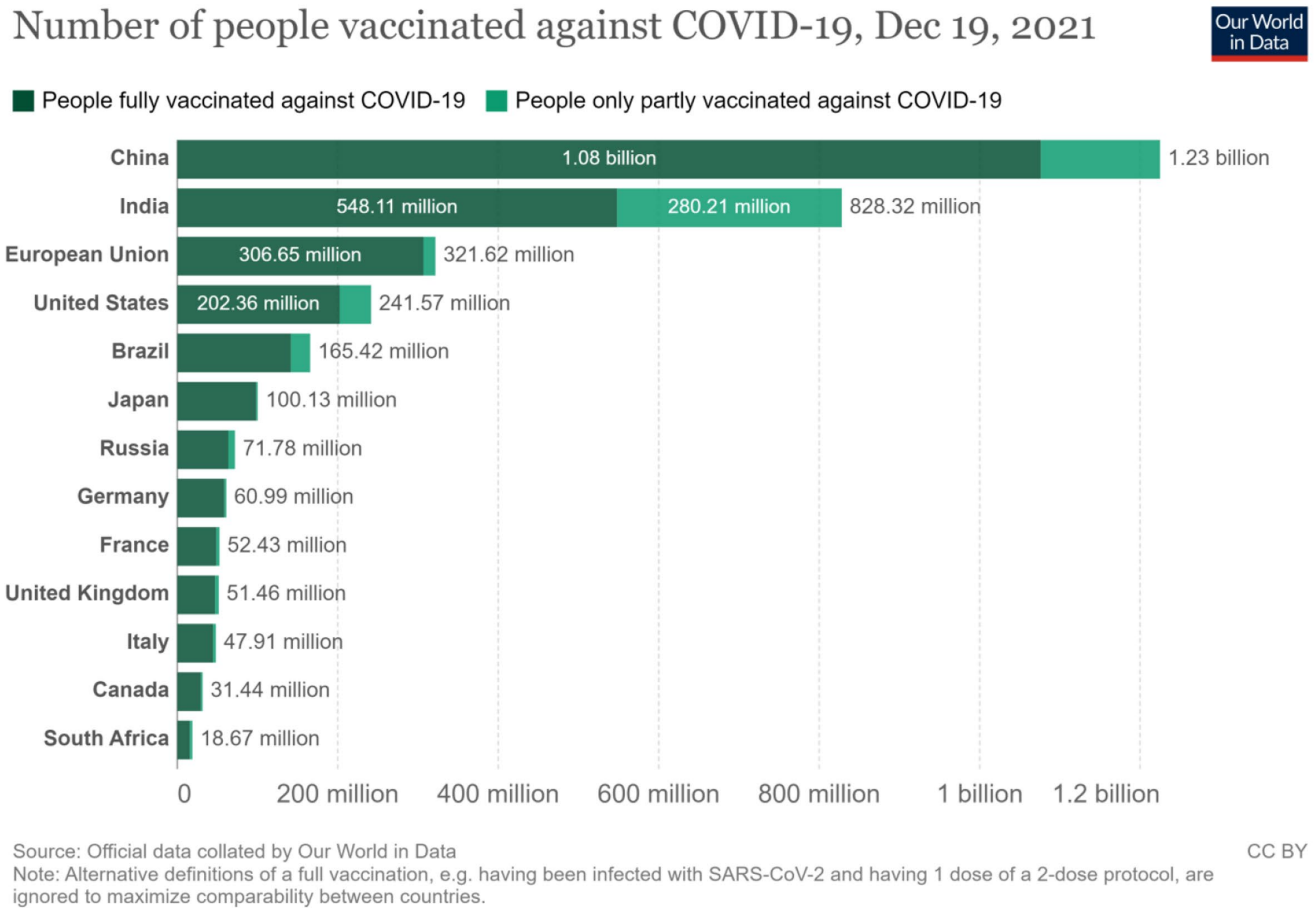
Number of persons vaccinated against COVID.

The information in Figure 6 compares the number of vaccinated people among the BRICS (Brazil, Russia, India, China, and South Africa) countries against those in the European Union, United States, and G7. This helps determine vaccine self-sufficiency’s production and vaccination delivery capacity components. The figure shows that the BRICS countries together have vaccinated 1,717.49 million people as of December 2021, whereas the European Union vaccinated 306.65 million people within this period. Similarly, the United States has vaccinated 202.65 million people, just as the G7 together had vaccinated 456.72 million people by the end of December 2019. In each case, most of the vaccines produced in the BRICS were home-based as opposed to those in the European Union, but generally, the information affirms the fact that the BRICS countries do not just have the production capacity, they also have a well-established vaccine distribution network that commensurates with the needs and challenges created by COVID-19.

**Figure 7:**
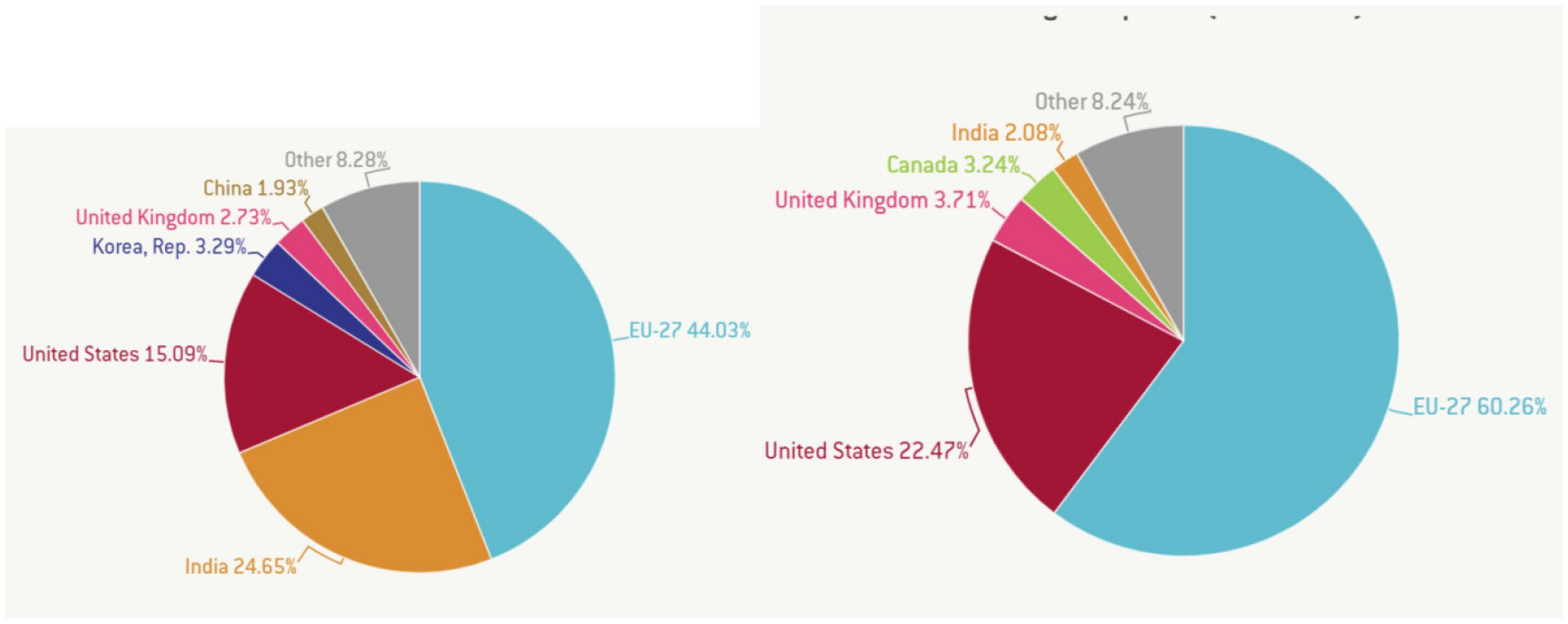
Largest exporters of vaccines by volume (left) and value (right). Source: Guetta-Jeanrenaud et al. (41) in Global Economy and Trade.

In Figure 7, the largest global exporters in the pharmaceutical industry are presented in terms of value and volume. The EU exports 27.44% of global vaccine needs, accounting for 27.60% of global pharmaceutical export value. India exports 24.65% of the global volume of pharmaceutical exports, representing 22.47% of the pharmaceutical export value. The other representative of the BRICS in this analysis is China, responsible for 1.93% of global pharmaceutical exports. Together, the BRICS countries are only accountable for approximately 24.64% of global vaccine exports but receive only 2.08% of their value.

## 4. Discussion

As mentioned above, in terms of subject matter, 11% of articles attributed the development of the BRICS vaccine industry to the innovation policies of these countries. Articles on the vaccine supply chain accounted for 15% of the total reviewed articles, 19% emphasized the catalytic role of Pharmaceutical production, 33% focused on vaccine production in BRICS countries, and 22% focused on health coordination among BRICS countries. In terms of research methods, 38% of the articles adopted qualitative research methods, and 9% adopted quantitative research methods. The rest of the paper adopts a mixed method. On this basis, this paper further extracts the core content of the selected articles for a more detailed review and analysis.

### 4.1. Entrepreneurial and innovation in vaccine self-sufficiency

Ezziane (21) traced the history of vaccine production in BRICS countries, claiming that vaccine self-sufficiency in BRICS countries does not have a long history, but considerable progress has been made over the past 20 years. According to Allen (25), this change is due to the fact that the BRICS countries have been extremely brave in changing themselves in order to promote self-sufficiency in vaccines and drugs. Zhou (42) revealed that the need to achieve vaccine self-sufficiency in BRICS countries was planned and implemented through public-private partnerships at the level of individual countries and inter-group cooperation. The public sector invests heavily in innovation by creating the conditions for innovation for proactive pharmaceutical entrepreneurs and large corporations.

This cooperation between the state and the private sector has helped individual countries to develop pharmaceutical entrepreneurs and companies willing to accept and introduce new pharmaceutical products and services through the innovation process and investment in research and development (6). Small pharmaceutical companies in the BRICS countries, in particular, have seen particularly significant growth by transferring ideas and inspiration from their contacts with the global pharmaceutical industry to investing in new technologies, supporting innovative practices, stimulating creative ideas, stimulating novelty, and persisting in experimentation to bring about new drug opportunities and new solutions to existing and new health challenges (28).

In some cases, BRICS governments have preferential purchase policies for domestic manufacturers and occasionally threaten compulsory licensing of medicines deemed essential for public health (33). These market and benchmark scientific risks for developing innovative health products in BRICS countries slow the rate at which large pharmaceutical companies make significant investments to push early-stage compounds through clinical trials and roll-out (43).

### 4.2. Artificial intelligence in drug development

The application of artificial intelligence to pharmaceutical products is one of the key factors that have grounded pharmaceutical production capability in the BRICS. Since 2005, several countries, including China, India, and Russia, have invested heavily in artificial intelligence and machine learning techniques to accelerate drug discovery (23). Both startups and large-scale pharmaceutical enterprises were encouraged to adopt AI-assisted automation, an optimization process for drug manufacturing, and marketing design to AI algorithms, which allow pharmaceutical companies in the BRICS to identify and recruit patients for clinical trials faster and more efficiently, which is a key step in the drug discovery and development process (29).

The growth of drug data analysis has also created pharmaceutical startups in China, India, and, in particular, Russia to analyze small and noisy data sets using different machine learning techniques (active learning, small amount learning, reinforcement learning, presentation learning), as well as deep learning solutions to predict and optimize potential drug candidates (24). This further eliminates the need for large data sets. As a result, AI technology simplifies and shortens the identification and eligibility criteria for clinical trials for vaccine production. The recruitment process for clinical trials has become faster, more rigorous, and less expensive, reducing lead time to the administration process without compromising on quality (34).

### 4.3. Big data analytics in drug development

Another crucial innovative capability in the BRICS that supports the drive for vaccine and pharmaceutical self-sufficiency is the application of Big Data and Analytics in the drug development process. Several modeling companies have emerged in the BRICS to take advantage of the volume of data generated during the drug discovery and development process (44). For example, between 2010 and 2020, 27 companies were registered in China and India to properly analyze data and derive value from drug manufacturing processes with high-performance systems to support new and existing drug manufacturing companies (45).

Through state-negotiated deals, pharmaceutical companies opened up their critical data to third parties with capabilities in advanced analytical techniques. This has turned historical and real-time data hitherto unused, underused, abused, or misused in the drug delivery process into critical competitive descriptive diagnostic, predictive, and predictive weapons for analyzing the collection of all medical data (patient records, hospital data, and medical imaging) to speed up and eliminate inefficiencies in drug development (6).

### 4.4. Flexible pharmaceutical production

The third source of innovation that has transformed the vaccine and pharmaceutical industry in the BRICS is the development of innovative solutions for flexible pharmaceutical production (46). From 2005, several BRICS countries began exploring new pharmaceutical manufacturing strategies like batches of precision medicine in response to changing market dynamics. Some IT companies emerged to offer single-use technology to eliminate complex steps, such as cleaning and validation between stages of production to reduce downtime and increase production (27).

### 4.5. Continuous manufacturing optimisation technologies

Another innovative technology introduced into the drug manufacturing processes in the BRICS is continuous manufacturing optimization technologies (26, 30, 47). For example, microfluidic droplet generators are emerging Microstructured elements that optimize and tailor the drug manufacturing process equipment down to an ideal scale. This technology improves and speeds up the intensified core pharmaceutical processes, such as crystallization, pervaporation, micro-encapsulation, and chemical synthesis (22).

### 4.6. Precision medicine and drug development

Precision medicine is one area that has also been enhanced in the quest to achieve vaccine and pharmaceutical self-sufficiency through efficient drug manufacturing practices in the BRICS. The concept of precision medicine helps to develop customized drugs to treat patients with unique pharmaceutical needs (32, 48). With advanced Omic studies and data analysis, new insights can be captured to better understand how an individual’s body reacts and responds to drugs. This is particularly helpful in additive manufacturing, which depends on drug exposure models to determine the pharmacodynamic and pharmacokinetic properties of drugs (49).

This further helps to settle on the accurate drug dosage for different clinical parameters such as age, gender, and comorbidities, as well as make real-time predictions on drug efficacy and interaction in specific individuals and groups of people, etc. This innovative capability is currently being explored to develop drug-specific exposure models for alternative vaccine formulas to cure COVID-19, which is currently being investigated in laboratories in BRIC countries (50). The innovative capacity of additive manufacturing is another important area that BRICS countries are exploring to achieve vaccine self-sufficiency (12).

### 4.7. Additive manufacturing

Since 2017, Chinese, Indian, and Russian pharmaceutical entrepreneurs have made a breakthrough in precision medicine research to launch advanced 3D bioprinters for printing tissues or cells. This initiative is critical in the drug development process, regenerative medicine, and organ engineering. This technology can facilitate the development of precision bills and medical formulations that depend on age and physiology (51). With these bio-printers, innovations in tissue engineering, bio-inks, and microfluidics can be scaled up.

These technologies are capable of making tissues have the desired structure and feature with a good cell survival rate, which was a major limitation in previous techniques used in testing new pharmaceutical products (50). Through electro-hydrodynamic printing techniques, new 3D bioprinters help deliver higher cell survival rates and tissues with micro and nano-scale features (12, 51–56). Thus, through pharmaceutical innovation, the BRICS countries have asserted their autonomy, boosting the quest for self-sufficiency (55).

### 4.8. Innovation policy and government support

Yueqin (56) explains that rejuvenated state support continues to play a significant role in the accelerated growth of the pharmaceutical manufacturing market in the BRICS. Aware of the changing dynamics of human society, each member of the bloc has developed and launched a national pharmaceutical innovation strategic plan to encourage innovation in the sector by the private sector (31). Most often, these strategies have arisen due to consistent, open, and frank public-private dialog and partnership. Despite the differences (timetable) in the depth and breadth of these policies, obvious similarities suggest that policymakers across the bloc have been learning from each other (36). Drug innovation at the 6th BRICS Summit (Brazil, 2014) was discussed and prioritized as a key feature of the drive for vaccine self-sufficiency (20, 37–40). At the summit, the unified geopolitical bloc discussed a mechanism to consciously leverage a new set of ideas, products, and values to capture the attention of the global health community (57). The parties agreed to challenge the status quo and lay the foundation for the systematic design of a range of innovative capabilities and opportunities for pharmaceutical companies and individuals to flourish through new market niches and product designs (38).

In addition, national regulatory authorities in these countries have been equipped with new technologies and experts to strengthen the vitality of national regulatory systems to monitor the production of more accurate drugs and vaccines, especially in China and India. For example, in 2011, the WHO successfully evaluated national regulatory authorities for vaccine clinical trials and certified them (5, 58). Recently, with the exception of South Africa, four regulatory authorities in BRICS countries have revised their regulatory functions to ensure compliance with WHO-prequalified vaccine targets. This assessment has facilitated the acceptance of many drugs produced in China on the international market and helped the development of the vaccine industry.

## 5. Conclusion

Over the past 20 years, the BRICS nations—Brazil, the Russian Federation, India, China, and South Africa—have achieved major strides in vaccine development, regulation, and production. The creation of the BRICS Vaccine Research and Development (R&D) Center will have a significant impact on vaccine cost and accessibility given the anticipated development of stronger research capability, production, and distribution technology, as well as stronger standardization to improve vaccine production quality in the near future (59). It is anticipated that the BRICS’ contributions to vaccine development will alter the global vaccination market and hasten the availability of vaccinations in developing nations. The challenge is turning these hopes into concrete plans of action and outcomes (60).

The vaccine is an important variety of biological medicine. Vaccines are automatic immune preparations made by artificially attenuated, inactivated, or genetically engineered pathogenic microorganisms (such as bacteria, viruses, etc.) and their metabolites to prevent infectious diseases (40). The vaccine industry consists of vaccine research and development, production, distribution, and vaccination. As can be seen from the above systematic review of innovation drivers, the current research on the BRICS vaccine industry has covered all aspects of the industry and adopted qualitative, quantitative, and mixed methods (58). The conclusions of the literature review indicate that direct state support, state support for pharmaceutical companies, and entrepreneurship and innovation have led to vaccine self-sufficiency in BRICS countries. Major entrepreneurial innovations that have accelerated BRICS vaccine self-sufficiency include investments in artificial intelligence (AI), big data analytics, and blockchain technology. These help speed up the drug delivery process by enhancing patient identification or optimizing potential drug candidates for clinical trials and production. Investments in flexible manufacturing techniques, continuous manufacturing optimization techniques, advances in precision medicine, and additive manufacturing technologies are making personalized medicine a reality (61).

However, it is worth pointing out that there are still some problems with the existing research. This is reflected in the following aspects: 1. Although there is an abundance of research on the vaccine industry, the number of studies on the BRICS countries is relatively small, which indicates that the academic circle’s attention on relevant issues needs to be further improved. In terms of the content of the articles included in the research, the vaccine industry itself, as a field of technological and political dynamic game, should not be ignored in the quantitative research on related issues. However, only 9% of the papers used quantitative research, and most of the papers used qualitative or a combination of qualitative and quantitative models.

## 6. Limitations

This review has some limitations that must be disclosed to avoid over-generalization of the conclusions. In order to avoid repeated entries of the same article affecting the accuracy of the research conclusions, only English articles from various major databases were selected for analysis. However, a significant number of BRICS countries do not speak English as their primary language, thus, it is likely that very important articles published in the original languages were excluded, and this may affect the generalization of the research.

Secondly, the study was restricted to articles published between January 2001 and December 2022. A wider timeline could bring additional information to improve the outcome of the research. For this reason, the influence of the time frame on the research conclusions may limit the interpretation of the results.

## Data availability statement

The original contributions presented in the study are included in the article/supplementary material, further inquiries can be directed to the corresponding author.

## Author contributions

YZ conceived the idea and revised the manuscript in line with the objectives. HL conceived the idea and sequentially aligned the parts of the research manuscript. XX analyzed the manuscript. HA drafted the manuscript. All authors read and approved the final manuscript.
